# The Incidence of Human Papillomavirus in Tanzanian Adolescent Girls Before Reported Sexual Debut

**DOI:** 10.1016/j.jadohealth.2015.10.248

**Published:** 2016-03

**Authors:** Catherine F. Houlihan, Kathy Baisley, Ignacio G. Bravo, Saidi Kapiga, Silvia de Sanjosé, John Changalucha, David A. Ross, Richard J. Hayes, Deborah Watson-Jones

**Affiliations:** a Clinical Research Department, London School of Hygiene and Tropical Medicine, London, United Kingdom; b Mwanza Intervention Trials Unit, Mwanza, Tanzania; c MRC Tropical Epidemiology Group, London School of Hygiene and Tropical Medicine, London, United Kingdom; d Unit of Infections and Cancer, Barcelona, Spain; e CIBER, Barcelona, Spain; f National Institute for Medical Research, Mwanza, Tanzania

**Keywords:** Human papillomavirus, Incidence, Sub-Saharan Africa

## Abstract

**Purpose:**

Acquisition of human papillomavirus (HPV) in women occurs predominantly through vaginal sex. However, HPV has been detected in girls reporting no previous sex. We aimed to determine incidence and risk factors for HPV acquisition in girls who report no previous sex in Tanzania, a country with high HPV prevalence and cervical cancer incidence.

**Methods:**

We followed 503 adolescent girls aged 15–16 years in Mwanza, Tanzania, with face-to-face interviews and self-administered vaginal swabs every 3 months for 18 months; 397 girls reported no sex before enrollment or during follow-up; of whom, 120 were randomly selected. Samples from enrollment, 6-, 12-, and 18-month visits were tested for 37 HPV genotypes. Incidence, clearance, point prevalence, and duration of any HPV and genotype-specific infections were calculated and associated factors were evaluated.

**Results:**

Of 120 girls who reported no previous sex, 119 were included, contributing 438 samples. HPV was detected in 51 (11.6%) samples. The overall incidence of new HPV infections was 29.4/100 person-years (95% confidence interval: 15.9–54.2). The point prevalence of vaccine types HPV-6,-11,-16, and -18 was .9%, .9%, 2.0%, and 0%, respectively. Spending a night away from home and using the Internet were associated with incident HPV, and reporting having seen a pornographic movie was inversely associated with HPV incidence.

**Conclusions:**

Incident HPV infections were detected frequently in adolescent girls who reported no previous sex over 18 months. This is likely to reflect under-reporting of sex. A low-point prevalence of HPV genotypes in licensed vaccines was seen, indicating that vaccination of these girls might still be effective.

**Implications and Contribution**

This study demonstrated that some girls who reported never having had sex had incident vaginal HPV, likely due to under-reporting of sex. However, 98% had no evidence of HPV-16 or -18, suggesting that catch-up vaccination campaigns in girls older than current WHO-recommended target population (9–13 years) may be effective at reducing cervical cancer.

Human papillomavirus (HPV) infects the mucosal surfaces of the female and male anogenital tract [1]. The predominant mechanism of HPV acquisition in women is thought to be through penetrative vaginal sex since historically, HPV has only been detected in a small proportion of girls and women who report no previous sex [2], [3], and HPV incidence and prevalence have been shown to increase rapidly in women after reported first sex [4]. Risk factors for incident HPV infection, identified in longitudinal data, include having a higher number of sex partners [5], [6]. In addition, HPV genotypes have been shown to be concordant between couples in sexual relationships [7]. High-risk (HR) HPV genotypes have been associated with cervical cancer [8], [9]. Vaccination against these HR HPV genotypes is recommended before first sex because the vaccine offers less protection once an HPV genotype has been acquired [10].

Tanzania has one of the highest rates of cervical cancer in the world and, to date, no national HPV vaccination program [11]. In a previous cross-sectional analysis, we identified a high prevalence (8.4%) of HPV in girls who reported no previous sex [12]. HPV detection was associated with reporting having cleaned inside the vagina, which may have been a marker for undisclosed sexual activity [13]. To further investigate the detection of vaginal HPV in girls who stated that they had not passed sexual debut, we present results from longitudinal follow-up of these girls in Mwanza, Tanzania. This study is the first to report the incidence of HPV in girls who report no previous sex in sub-Saharan Africa.

## Methods

### Study procedures

The cohort was enrolled between January and August 2012, as described previously [12]. Briefly, we enrolled eligible girls attending government primary schools in three districts in the Mwanza region of Tanzania that had been randomly selected in preparation for an HPV vaccine trial [14]. Eligibility criteria included having been in Class 6 in 2010 in one of the nonvaccine schools, reporting never having previously had sex, being willing to undergo study procedures, able to attend appointments, and being 15 or 16 years old at the time of enrollment.

During the study, samples were collected every 3 months for 18 months as described previously [12]. At enrollment and each follow-up visit, girls underwent a face-to-face interview in Swahili using a structured questionnaire, which included questions on sexual behavior and intravaginal practices. Girls were additionally asked to provide one self-administered vaginal swab under the supervision of a trained research nurse. We present data from a randomly selected subgroup of girls who reported not having had sex during study follow-up.

### Ethical considerations permissions

The Medical Research Coordinating Committee, Tanzania (Ref: NIMR/HQ/R.8a/Vol. IX/1249) and the London School of Hygiene and Tropical Medicine Ethics Committee (Ref: 6040) approved the study protocol. Because participants were younger than the age of legal consent, written informed consent was required from a parent/guardian before written participant assent. Girls with persistent HR HPV at study completion (defined as the same HR HPV genotype detected at two consecutive 6-month visits) were referred for follow-up with the National Cervical Screening Program.

### Human papillomavirus detection and genotyping

Dry vaginal swabs were stored in cryotubes immediately after collection and transported in cold boxes with ice packs before submission to the Mwanza Intervention Trials Unit Laboratory in Mwanza, where they were stored at −20°C until they were shipped to the Catalan Institute of Oncology in Barcelona. Samples from enrollment, month-6, -12, and -18 visits for girls included in this substudy were tested using the Linear Array HPV genotyping assay (Roche, CA) which detects 13 HR and 24 low-risk (LR) genotypes [15]. We considered HPV genotypes categorized by the International Agency for Research on Cancer as associated with cancer (Group I) and probably associated with cancer (Group IIa) as HR HPV genotypes [16]. All other genotypes (Groups IIb: possibly associated with cancer and III: not associated with cancer) were considered LR types. Samples negative for β-globin were considered inadequate for HPV genotyping.

### Data management and statistical methods

Data were double entered into OpenClinica LLC (Akaza Research, MA), and analysis was performed using Stata version 10.3 (StataCorp LP, TX).

Reported sexual behaviors were tabulated in all girls who reported not having had sex and in those included in the present study. Genotype-specific incidence was calculated using person-time at risk from the date of enrollment. A new (incident) infection was defined as a first positive test for the specific HPV type among those not infected with that genotype at enrollment. Date of infection was assumed to be midway between the last negative and first positive sample. Girls who had missed visits or missing HPV results, which led to a gap in the observations, were censored at the last available result before the gap.

Duration of infection was calculated from the date of infection to the date of clearance (assumed to be midway between the last positive and first negative sample for that genotype). Clearance of a genotype-specific infection was defined as a single negative sample for that genotype. Infections that were not cleared were censored at the date of the last positive sample. If there were two positive samples with one intervening missing sample (e.g., positive at month-6 and -18 but with a missing sample at month-12), duration was considered unknown and censored at the date of the first positive sample.

The overall incidence of all HPV infections was calculated as the number of new infections (allowing multiple events at each time point) over person-years at risk. Gaps (explained previously) where a visit was missed were removed, but girls were assumed at risk again after a gap. The overall incidence rate was estimated using Poisson regression with random effects to account for clustering of infections within the same girl. The HPV genotype–specific point prevalence was estimated as the number of visits where the genotype was detected, divided by the total number of visits, including enrollment.

Poisson regression with random effects was used to estimate rate ratios for factors associated with HPV incidence. A conceptual framework with three levels was used to build an adjusted model; age was considered an a priori confounder. Age-adjusted sociodemographic factors at enrollment were retained in a core model if associated with HPV infection at *p* < .10. Time-varying sociodemographic factors were added to this core model sequentially and retained if associated at *p* < .10. Time-varying behavioral factors were then added sequentially and retained if they remained associated at *p* < .10. This strategy allowed us to assess the effects of variables at each level of the framework, adjusted for more distal variables.

## Results

Study enrollment has been described previously [12]. Of 628 girls located, age eligible, and consented, 504 (80%) reported never having had sex, and 481 were enrolled. Of those enrolled, 397 reported never having had sex during the study and confirmed this at the final study visit. We randomly selected 120 (30.2%) of these girls and excluded one girl who spontaneously reported that she had been HIV positive from birth (girls were not explicitly asked this). Table 1 summarizes participant characteristics and reported behaviors in all 396 girls in the cohort who reported no previous sex and in the 119 girls included in this analysis.

At enrollment, 61 of the 119 (51.3%) girls were aged 16 years, and the remainder was aged 15 years. Approximately half (52.9%) lived in urban areas, 92.4% were Christian, 79.0% were in school, and 19.3% were neither working nor in school. The final study visit was attended by 90.8% of girls. At this visit, 108 girls were asked if they were circumcised (i.e., had experienced genital cutting), and none reported this. Girls contributed 438 valid specimens.

Overall, 10 of the 119 girls (8.5%) who never reported having had sex had HPV detected at enrollment. The 119 girls contributed a total of 152.8 person-years (pys) of follow-up; during which, 44 new HPV infections were detected. The overall incidence of new HPV infections in girls who reported never having had sex was 29.4 per 100 pys (95% confidence interval [CI]: 15.9–54.2) and for new HR HPV infections was 11.3/100 pys (95% CI: 5.8–22.1; Table 2). Infection with at least one HPV genotype was detected at 11.6% of visits, and at least one HR HPV genotype was detected at 6.6% of visits.

**Figure 1:**
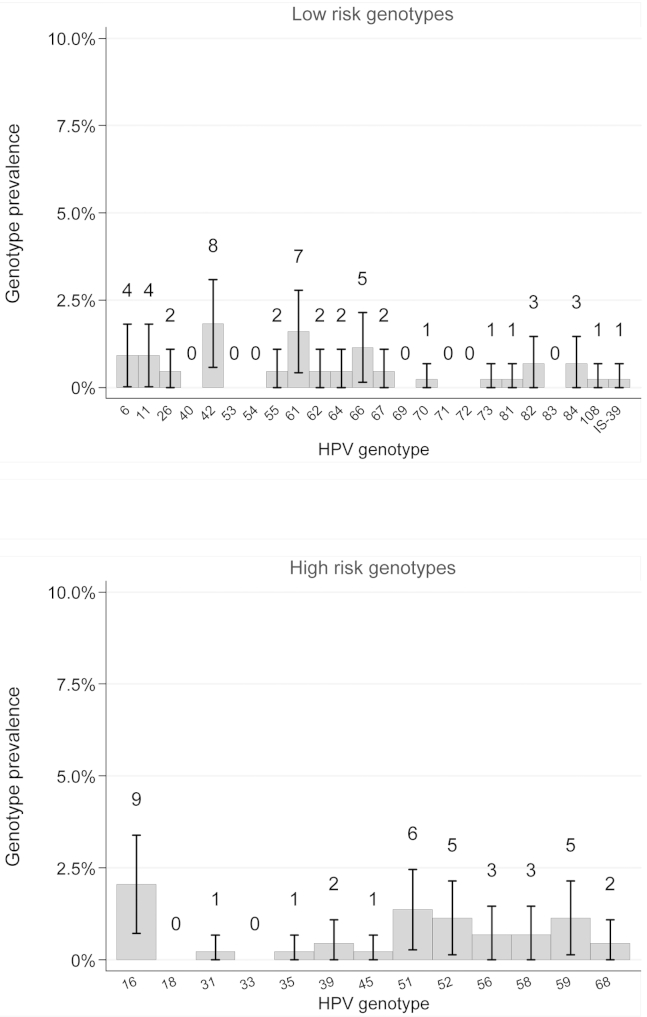
HPV-genotype point prevalence (95% CI) and number of infections at all visits including enrollment among 119 girls who did not report sex at enrollment or during 18-month follow-up. The HPV genotype-specific point prevalence was estimated as the number of visits where the genotype was detected, divided by the total number of visits attended including the enrollment visit. Visits with missing vaginal samples, or with samples that were β-globin negative, were excluded.

The HPV genotype with the highest incidence was HPV66 (2.7/100 pys), followed by HPV59 (2.6/100 pys), and HPV-16, -51, -52, and -6 (2.0/100 pys for each; Table 3). The genotypes with the highest point prevalence were HPV-16 (2.1%), HPV-42 (1.8%), and HPV-61 (1.6%; Figure 1).

The mean duration of any new HPV infection was 7.0 months; 8.1 for any HR HPV infection, and 6.6 for any LR infection (Table 3). The rate of clearance of LR HPV infections was three times more than that of HR HPV infections (103.3 vs. 28.8/100 pys).

In the adjusted analysis, there was strong evidence that having spent a night away from home in the last 6 months (adjusted risk ratio [aRR] = 5.47; 95% CI = 1.72–17.4) or having used the Internet (aRR = 3.90; 95% CI = 1.05–14.50) was associated with new HPV infection (Table 4). There was weak evidence that having watched a pornographic movie was associated with a lower risk of HPV acquisition (aRR = .18; 95% CI = .03–1.03) and that having cleansed inside the vagina with soap in the past 6 months, compared with not having cleansed was associated with a new HPV infection (aRR = 2.64; 95% CI = .75–9.31).

## Discussion

A total of 119 Tanzanian adolescent girls who reported never having sex were followed for 18 months. Tanzania has a very high HPV prevalence and one of the world's highest incidences of cervical cancer [11], [17]. We found that 11% of the samples provided during the study period showed evidence of vaginal HPV infection, which likely reflects under-reporting of sex or, less likely, acquisition of HPV through nonpenetrative sex or vaginal cleansing. Studies have compared self-reporting of sex with biomarkers of recent sex [18], compared different methods of interview [19], [20], or carried out repeat surveys [21] in East Africa, and have shown that under-reporting of sex is common. Under-reporting may be particularly common in adolescent girls in Tanzania since sex outside marriage or while schooling may result in school expulsion, physical punishment, or legal reprimand for the male partner [22], [23].

A number of cross-sectional studies have found that cervical or vaginal HPV was detected in 0%–1.5% of self-reported virgins in Europe, Australia, and United States of America [2], [3], [24], [25]. Longitudinal studies, which sampled girls who consistently reported no previous sex at multiple time points, have detected higher rates of HPV. One study in girls in the United States of America, median age of 19 years, detected HPV in 7.8% of girls who were followed for 24 months and consistently reported that they had never had sex [4]. Another study in the United States of America followed 14- to 17-year-old girls every 3 months for a median of 5.2 years and found HPV in 10 of 22 (45.5%) who reported never having had sex [26]. Both studies identified an association between nonpenetrative sexual behaviors and HPV detection in girls. Nonpenetrative sex practices including hand–genital contact and oral–genital sex have been identified as risk factors for HPV acquisition in heterosexual women [4], [27] and in homosexual women who report never having had penile–vaginal sex [28]. In our study population, oral–genital sex is infrequently reported in adolescents [22], [29] and was not reported by any of our participants. Hand–genital contact was reported by only two participants.

Intravaginal cleansing was associated with prevalent HPV in girls who reported never having had sex at the time of cohort enrollment [12]. We have suggested that intravaginal cleansing was most likely to have been a marker for unreported sex but additionally commented that the practice of intravaginal cleansing, which involves inserting fingers or a cloth inside the vagina, may introduce HPV from external genitalia or fomites. In this current longitudinal study, there was no strong evidence that recent intravaginal cleansing was associated with new HPV infection. This may be due to the more robust analysis offered by longitudinal data compared with cross-sectional data. A further possible explanation may be the small number of girls (n = 6) who at enrollment reported no previous sex but corrected this during follow-up; these girls were included in the cross-sectional analysis [12] but excluded from this study. There was weak evidence of an association with reported cleansing with soap compared with not cleansing in the past 6 months. Again, this is most likely to be a marker of unreported sex because girls may be more likely to perform intravaginal cleansing if they believe that this practice is appropriate or desirable before or after sex or that it is effective in reducing the risk of STI or pregnancy [13]. HPV has been detected on fingers of women with genital warts, on toilet seats in airports, and on surfaces in sexual health clinics in the United Kingdom [30], [31], [32], and therefore self-infection, via fingers or fomites (e.g., cloths), remains a theoretical possibility.

We also observed a strong association between spending a night away from home in the past 6 months and HPV acquisition. Traveling or short-time migration has been associated with HIV infection in previous studies in sub-Saharan Africa [33], [34], but not with HPV. It is likely that spending a night away from home is a marker for unreported sex and therefore HPV risk.

One of the limitations of this study was that we only tested samples from girls every 6 months. Therefore, it is likely that HPV-incidence was underestimated because some short-lived infections may have cleared quickly between visits. This may have been partially mitigated by using the highly sensitive HPV Roche Linear Array, reducing the risk of false-negative tests. A further limitation of the study is the relatively small number of events in the risk factor analysis, which resulted in large standard errors and reduced power to detect associations, and made it difficult to adequately adjust for all potential confounders. The major strength of this study was the face-to-face interviews. These included current colloquial terms for sexual behaviors. The interviewers were experienced in sexual behavior research with adolescents and attempted to meet the same participant at every visit, establishing a trusting relationship and potentially reducing under-reporting. A nested study of alternative interview methods found no increase in reporting of sex. Unfortunately, interviews did not include questions about masturbation, which has previously been reported by adolescent girls in Tanzania [29] and which could potentially lead to transmission via fingers. Similarly, HPV could be acquired through fomite transmission during female genital cutting via unsterilized equipment or the fingers of the practitioner. Although only one participant in our cohort reported having undergone genital cutting, this may have been an underestimate: a study of 15- to 44-year-old women in Tanzania found 73% had evidence of genital cutting [35]. Finally, this cohort were selected based on reporting never having had sex and were recruited from school registers, potentially affecting representativeness. However, attendance at primary school is a legal requirement, and most children are likely to at least be registered in primary school. Furthermore, of those located and age eligible, 80% reported never having had sex, indicating that the cohort is likely to be representative of most girls of the same age who are enrolled in schools in Tanzania.

Our results demonstrate that some Tanzanian adolescent girls who report never having had sex have a relatively high incidence of vaginal HPV. HPV-16 and -18 are responsible for >70% of cervical cancers, and currently available HPV vaccines offer a high level of protection from these genotypes [36], [37]. In our study, HPV-16 was the most frequently detected genotype. However, 98% of these girls had no evidence of HPV-16 or -18, suggesting that catch-up vaccination campaigns in girls older than the current target population recommended by WHO (9–13 years old [38]) may be effective at reducing cervical cancer. This may be particularly relevant because the Tanzania vaccination program has proposed primarily targeting girls in Year 4 if they are in school or 9-year-old girls if out of school.

## Figures and Tables

**Table 1:** Prevalence of reported behaviors among all girls in the study who did not report sex at enrollment or during follow-up (n = 396) and among those randomly selected for the HPV incidence study (n = 119)

Reported behavior	Number of girls reporting the behavior among all (n = 396) who did not report sex^a^ (%)	Number of girls reporting behavior among (n = 119) who did not report sex^a^ and who have HPV results (%)
At enrollment		
Ever drank alcohol		
Yes	1 (.3)	0 (0)
Ever smoked		
Yes	0 (0)	0 (0)
Ever took drugs		
Yes	0 (0)	0 (0)
Ever-cleansed vagina		
Yes	68 (17.2)	20 (16.8)
Ever-kissed a boy		
Yes	0 (0)	0 (0)
Boy ever touched breasts		
Yes	22 (5.6)	8 (6.7)
Ever touched boy's genitals or boy touched girl's genitals		
Yes	2 (.5)	0 (0)
Ever had oral sex		
Yes	0 (0)	0 (0)
During follow-up^b^		
Spent a night away since last visit		
Yes	272 (68.7)	86 (72.3)
Used Internet ever^c^		
Yes	95 (24.0)	35 (29.4)
Ever watched a pornographic movie^c^		
Yes	62 (15.7)	17 (14.3)
Drank alcohol since last visit		
Yes	4 (1.0)	1 (.8)
Practiced vaginal cleansing since last visit		
Yes	141 (35.6)	42 (35.3)
Kissed a boy since last visit		
Yes	6 (1.5)	3 (2.5)
Boy touched breasts since last visit		
Yes	12 (3.0)	3 (2.5)
Touched boy's genitals/boy touched girl's genitals since last visit		
Yes	2 (.5)	1 (.8)

a Girls who did not report sex at enrollment or during the study up to and including the final visit (18 months).

b Girls were asked at every visit whether they had experienced any of these behaviors since they were last seen in the study.

c Girls were asked during follow-up if they had ever used the Internet or seen a pornographic movie, but they were not asked when they had done that. After a girl reported ever having used the Internet or having seen a pornographic movie, all subsequent visits are counted as “yes.”

**Table 2:** Incidence and point prevalence in adolescent girls who did not report sex at enrollment or during 18 months follow-up

Outcome	All girls (N = 119)^a^	Negative for all HPV genotypes at enrollment (HPV naïve; N = 109)^b^
Incidence	New infections/person-years (rate/100 person-years, 95% CI)^c^	
All HPV	44/152.8 (29.4; 15.9–54.2)	40/140.6 (27.9; 14.7–53.0)
All HR HPV	18/152.8 (11.3; 5.8–22.1)	18/140.6 (12.4; 6.4–24.0)
All LR HPV	26/152.8 (18.1; 9.3–35.1)	22/140.6 (15.8; 7.9–31.7)
Prevalence	Total infections (number of visits with at least one infection/all visits; % of all visits)^d^	
All HPV	87 (51/438; 11.6)	50 (24/402; 6.0)
All HR HPV	38 (29/438; 6.6)	23 (16/402; 4.0)
All LR HPV	49 (36/438; 8.2)	27 (17/402; 4.2)

CI = confidence interval; HPV = human papillomavirus; HR = high-risk; LR = low-risk.

a HPV incidence among all girls who did not report passing sexual debut during the study includes 10 girls in whom prevalent HPV was detected at enrollment.

b HPV incidence among girls who did not report passing sexual debut during the study and no HPV was detected at enrollment.

c Rate estimated from random effects Poisson regression: point estimates and 95% CI take into account correlation of repeated events within girls. Girls assumed to be continually at risk and can acquire more than one infection at each visit.

d Total number of genotype-specific infections and number of visits where at least one genotype was detected, at all visits including enrollment visit.

**Table 3:** HPV genotype prevalence, incidence, duration, and clearance among 119 girls who did not report sex at enrollment or during up to 18 months follow-up

HPV type	Prevalent HPV infections (%)^a^	New infections/pys (rate/100 pys)^b^	New infections that were cleared (%)^c^	Number of new infections cleared/pys (rate/100 pys)	Mean (median) months duration (Kaplan–Meier)^d^
HR genotypes					
HPV-16	1 (1)	3/149.7 (2.0)	0	0/1.7 (0)	9.2^e^ (f)
HPV-18	0	0/152.8 (0)	—	—	—
HPV-31	0	1/152.6 (.7)	0	0/.3 (0)	g
HPV-33	0	0/152.8 (0)	—	—	—
HPV-35	1 (1)	0/151.4 (0)	—	—	—
HPV-39	0	1/152.1 (.7)	0	0/.7 (0)	g
HPV-45	0	0/152.8 (0)	—	—	—
HPV-51	1 (1)	3/150.6 (2.0)	2 (67)	2/1.3 (159.0)	5.9 (5.8)
HPV-52	1 (1)	3/150.6 (2.0)	0	0/.8 (0)	3.2^e^ (f)
HPV-56	1 (1)	1/152.1 (.7)	0	0/.3 (0)	g
HPV-58	2 (2)	1/151.1 (.7)	0	0/.2 (0)	g
HPV-59	1 (1)	4/151.3 (2.6)	0	0/1.0 (0)	3.1^e^ (f)
HPV-68	0	1/152.2 (.7)	0	0/.7 (0)	g
** All HR infections**^h^	**8**	**18**	**2 (11)**	**2/6.9 (28.8)**	**8.1**^e^**(**f**)**
LR genotypes					
HPV-6	0	3/151.1 (2.0)	1 (33)	1/1.5 (67.3)	7.5^e^ (6.0)
HPV-11	1 (1)	1/150.2 (.7)	1 (100)	1/.5 (194.3)	g
HPV-26	0	2/151.4 (1.3)	1 (50)	1/.7 (135.0)	5.8 (5.8)
HPV-40	0	0/152.8 (0)	—	—	—
HPV-42	2 (2)	1/150.1 (.7)	0	0/.3 (0)	g
HPV-53	0	0/152.8 (0)	—	—	—
HPV-54	0	0/152.8 (0)	—	—	—
HPV-55	0	1/152.1 (.7)	0	0/.8 (0)	g
HPV-61	1 (1)	2/150.4 (1.3)	1 (50)	1/.7 (136.5)	5.8 (5.8)
HPV-62	1 (1)	1/150.7 (.7)	1 (100)	1/.5 (207.5)	g
HPV-64	0	2/151.9 (1.3)	1 (50)	1/.7 (137.6)	5.8 (5.8)
HPV-66	0	4/150.9 (2.7)	1 (25)	1/1.7 (58.0)	7.5^e^ (5.8)
HPV-67	0	2/150.9 (1.3)	2 (100)	2/1.0 (207.5)	5.8 (5.8)
HPV-69	0	0/152.8 (0)	—	—	—
HPV-70	0	1/152.6 (.7)	0	0/.3 (0)	g
HPV-71	0	0/152.8 (0)	—	—	—
HPV-72	0	0/152.8 (0)	—	—	—
HPV-73	0	1/152.6 (.7)	0	0/.2 (0)	g
HPV-81	1 (1)	0/151.4 (0)	—	—	—
HPV-82	1 (1)	1/152.1 (.7)	0	0/.3 (0)	g
HPV-83	0	0/152.8 (0)	—	—	—
HPV-84	1 (1)	2/150.3 (1.3)	1 (50)	1/.7 (135.3)	6.0 (6.0)
HPV CP-108	0	1/152.6 (.7)	0	0/.2 (0)	g
HPV IS-39	0	1/152.1 (.7)	1 (100)	1/.5 (208.1)	g
** All LR infections**^h^	**8**	**26**	**11 (42)**	**11/10.6 (103.3)**	**6.6**^e^**(5.8)**
**All HPV infections**	**16**	**44**	**13 (30)**	**13/17.6 (73.9)**	**7.0**^e^**(6.0)**

Overall figures are highlighted in bold.

HPV = human papillomavirus; HR = high-risk; LR = low-risk; pys = person-years.

a Positive for that genotype at the enrollment visit.

b New infection defined as first positive test for the specific HPV type, among those not infected at enrollment.

c Clearance defined as a negative sample for the specific genotype; denominator is total genotype-specific new infections.

d Mean duration of new infections estimated using Kaplan–Meier methods restricted by the longest follow-up time (i.e., duration).

e Mean duration of infection for the genotype is underestimated because the individual with the longest observed duration was censored.

f Median duration could not be estimated because survival curve does not drop below 50%.

g One infection only, Kaplan–Meier survival function not estimated.

h Total number of group (HR or LR)-specific infections among 119 girls.

**Table 4:** Factors associated with any new HPV infection among 119 girls who did not report sex at enrollment or during 18 months follow-up

Variable	Number of infections/person-years (rate/100 pys)	Crude RR (95% CI)	Adjusted RR (95% CI)^a^
Sociodemographic at enrollment			
Age at enrollment		*p* = .40	
15 years	29/73.9 (36.9)	1	
16 years	15/78.9 (21.7)	.59 (.17–1.99)	
Religion		*p* = .78	*p* = .85
Christian	37/141.5 (27.2)	1	1
Muslim	5/8.9 (50.0)	1.84 (.17–19.81)	1.70 (.16–18.03)
Other	2/2.4 (69.9)	2.57 (.04–176.6)	2.08 (.03–137.8)
SES score (tertiles)^b^		*p* = .56	*p* = .76
Low	9/47.1 (19.3)	1	1
Middle	12/52.4 (25.0)	1.30 (.27–6.13)	1.21 (.24–6.02)
High	23/53.4 (42.4)	2.20 (.49–9.81)	1.89 (.32–11.12)
Sociodemographic (time varying)			
Current residence		*p* = .07	*p* = .09
Urban	34/84.7 (40.3)	1	1
Rural	10/68.1 (15.9)	.39 (.14–1.10)	.41 (.15–1.16)
Current occupation		*P* = .24	*p* = .24
School	28/117.5 (27.2)	.27 (.02–2.89)	.30 (.03–3.05)
Work/vocational training	1/7.8 (7.2)	1	1
Not working	15/27.6 (43.9)	1.61 (.65–4.02)	1.69 (.68–4.24)
Spent a night away since last seen		*p* < .001	*p* < .001
No	26/112.0 (20.0)	1	1
Yes	18/40.8 (109.6)	5.47 (1.72–17.4)	5.63 (1.81–17.49)
Behavioral (time varying)			
Current menstrual hygiene		*p* = .12	*p* = .18
Cloth	10/58.6 (22.5)	1	1
Underwear	8/21.6 (21.8)	.97 (.23–4.10)	.72 (.15–3.56)
Sanitary pad	26/60.3 (45.3)	2.02 (.55–7.34)	1.31 (.30–5.76)
Premenarche	0/12.4 (.0)	—	—
Used Internet^c^		*p* = .12	*p* = .05
No	34/132.7 (25.9)	1	1
Yes	10/20.1 (58.0)	2.24 (.79–6.36)	3.08 (.94–10.13)
Watched a pornographic movie^c^		*p* = .20	*p* = .05
No	42/137.9 (31.6)	1	1
Yes	2/15.0 (11.5)	.36 (.07–1.92)	.22 (.04–1.24)
Cleansed vagina in last 6 months^d^		*p* = .03	*p* = .08
No	24/118.4 (21.2)	1	1
Yes	20/34.4 (59.2)	2.79 (1.08–7.17)	2.43 (.87–6.76)
Substance used for vaginal cleansing		*p* = .01	*p* < .001
Did not cleanse	30/126.8 (25.5)	1	1
Soap or soap and water	14/19.1 (103.4)	4.05 (1.30–12.63)	3.01 (.86–10.59)
Water only	0/6.9 (.0)	—	—
Kissed a boy in the last 6 months		*p* = .56	*p* = .36
No	43/151.4 (28.6)	1	1
Yes	1/1.5 (87.1)	3.05 (.06–155.9)	5.88 (.09–390.8)
Boy touched participant's breasts in last 6 months			
No	44/151.9 (29.6)	—	—
Yes	0/1.0 (.0)	—	—
Boy touched participant's genitals or participant touched boy's genitals in the last 6 months			
No	44/152.4 (29.4)	—	—
Yes	0/.5 (.0)	—	—

CI = confidence interval; pys = person-years; RR = relative risk.

a Sociodemographic factors at enrollment adjusted for age (a priori). Time-varying sociodemographic factors adjusted for age (a priori); no other baseline sociodemographic factors were associated with HPV infection. Time-varying behavioral factors adjusted for age (a priori) and all independent sociodemographic factors: spending a night away.

b Socioeconomic status (SES) was determined using an asset index created by combining data collected from the entire cohort at enrollment on household ownership of the following items: radio, phone, car, motorbike, bicycle, livestock, television, or land. Principal components analysis techniques were used.

c Girls were asked during follow-up if they had ever used the Internet or seen a pornographic movie, but they were not asked when they had done that. After a girl reported ever having used the Internet or having seen a pornographic movie, all subsequent visits are counted as “yes.”

d Vaginal cleansing is cleaning inside the vagina with water, soap, or other products using fingers or a cloth.
